# PTBP1 knockdown promotes neural differentiation of glioblastoma cells through UNC5B receptor

**DOI:** 10.7150/thno.71100

**Published:** 2022-05-09

**Authors:** Kankai Wang, Sishi Pan, Peiqi Zhao, Li Liu, Zhen Chen, Han Bao, Hao Wang, Ying Zhang, Qichuan Zhuge, Jianjing Yang

**Affiliations:** 1 Zhejiang Provincial Key Laboratory of Aging and Neurological Disorder Research, The First Affiliated Hospital of Wenzhou Medical University, Wenzhou 325000, China.; 2 Department of Neurosurgery, The First Affiliated Hospital of Wenzhou Medical University, Wenzhou 325000, China.; 3 Zhejiang Provincial Key Laboratory of Interventional Pulmonology, The First Affiliated Hospital of Wenzhou Medical University, Wenzhou 325000, China.

**Keywords:** glioblastoma, cell reprogramming, *PTBP1*, proliferation, neural differentiation

## Abstract

**Rationale:** Cell reprogramming technology is utilized to prevent cancer progression by transforming cells into terminally differentiated, non-proliferating states. Polypyrimidine tract binding protein 1 (PTBP1) is an RNA binding protein required for the growth of neurons and may directly transform multiple normal human cells into functioning neurons *in vitro* and *in vivo* when expressed at low levels. As a result, we identified it as a key to inhibiting cancer cell proliferation by boosting glioblastoma cell neural differentiation.

**Methods:** Immunocytofluorescence (ICF) targeting TUJ1, MAP2, KI67, and EdU were utilized to evaluate glioblastoma cell reprogramming under *PTBP1* knockdown or other conditions. *PTBP1* and other target genes were detected using Western blotting and qRT-PCR. Activating protein phosphatase 2A (PP2A) and RhoA were detected using specific kits. CCK8 assays were employed to detect cell viability. Bioluminescence, immunohistofluorescence (IHF), and Kaplan-Meier survival analyses were utilized to demonstrate the *in vivo* reprogramming efficiency of *PTBP1* knockdown in U87 murine glioblastoma model. In this study, RNA-seq technology was used to examine the intrinsic pathway.

**Results:** The expression of TUJ1 and MAP2 neural markers, as well as the absence of KI67 and EdU proliferative markers in U251, U87, and KNS89 cells, indicated that glioblastoma cell reprogramming was successful. *In vivo*, U87 growth generated xenografts was substantially shrank due to *PTBP1* knockdown induced neural differentiation, and these tumor-bearing mice had a prolonged survival time. Following RNA-seq, ten potential downstream genes were eliminated. Lentiviral interference and inhibitors blocking tests demonstrated that UNC5B receptor and its downstream signaling were essential in the neural differentiation process mediated by *PTBP1* knockdown in glioblastoma cells.

**Conclusions:** Our results indicate that *PTBP1* knockdown promotes neural differentiation of glioblastoma cells via UNC5B receptor, consequently suppressing cancer cell proliferation *in vitro* and *in vivo*, providing a promising and feasible approach for glioblastoma treatment.

## Introduction

The World Health Organization (WHO) announced three classifications of adult-type diffuse gliomas in 2021 based on isocitrate dehydrogenase (*IDH*) mutation and 1p19q co-deletion status 1. Glioblastoma (*IDH*-wildtype) accounts for more than 70% of all occurrences, with a prevalence rate of 3.23 cases per 100,000 person-years 2, 3. The median overall survival time (OS) of newly diagnosed glioblastoma patients is less than two years under the current accepted therapeutic scheme, including accurate resection, prompt radiation, and suitable chemotherapy 4. Despite significant research advancement in various aspects, such as the discovery of risk gene-targeted drug delivery systems, as well as imaging technologies that make it easier for surgeons to perform surgeries, it remains difficult to change the prognosis of glioblastoma patients 5-8.

Recently, high-efficiency cell conversion has been achieved by reprogramming the genes and transcription factors that determine cell differentiation 9-11. Polypyrimidine tract binding protein 1 (PTBP1), an RNA-binding protein consisting of an N-terminal nuclear shuttling domain and four quasi-RRM domain repeats 12, has been demonstrated to transform numerous cultivated cells into functioning neurons when expressed at low levels 13, 14. Even adenoviruses that knockdown *PTBP1* have been indicated to promote glia into functioning neurons *in situ* to replace damaged or dysfunctional cell networks 15, 16. Although the effect of *PTBP1* on glioblastoma cell proliferation has been reported several times, Cheung et al. reported that knocking down *PTBP1* can alter the selective splicing of reticulon, influencing glioblastoma cell proliferation and invasion 17-22. What we propose, however, is a method that can transform unrestricted cancer cells into a terminally differentiated, non-proliferating state *in vitro* and *in vivo*.

In this research, we found that *PTBP1* knockdown promoted neural differentiation in U251, U87 and KNS89 human glioblastoma cells and significantly decreased their proliferation *in vitro*. Besides, *PTBP1* knockdown U87 cells grew significantly slower due to successful reprogramming *in vivo*. In addition, after *PTBP1* knockdown, RNA-seq was employed to detect variations in mRNA in U251 cells, followed by enrichment analysis. Ten potential downstream genes were identified, and their effects on glioblastoma cell reprogramming were investigated *in vitro* using lentiviral interference or compounds. These findings validated the role of UNC5B receptor and its downstream signaling in *PTBP1* knockdown-induced reprogramming process.

## Materials and Methods

### Animals and ethics consideration

The Shanghai Charles River Experimental Animal Limited Liability Company (Shanghai, China) provided nude mice weighing 20 g. These animals were housed in controlled environments with free access to food and water. The Animal Ethics Committee of Wenzhou Medical University approved all experimental procedures carried out in strict conformity with the National Institutes of Health's animal care and use guidelines.

### Model and imaging system for a brain tumor

Nude mice (n = 10) were anesthetized by isoflurane (2%) and positioned on the stereotaxic apparatus (RWD Life Science, China). According to animal surgery regulations, a 2 mm diameter bone window (1 mm before the sagittal suture, 2 mm to the right of the midline) was exposed for injection. A micro-syringe was used to inject U87 cells (500,000/5 µL) into the striatum (3 mm below the dura) three days after infection. The bone hole was sealed with bone wax after injection, and operators sutured the scalp. Following that, the mice were typically housed. The growth of tumor mass (n = 5) was evaluated every seven days using PerkinElmer IVIS Lumina X5 (USA) *in vivo* imaging system (captured 10 min after luciferin injection with a 10-second exposure duration), and the date of natural death was documented for survival analysis 23. In addition, five mice from each group were euthanized, and IHF samples were taken 28 days after transplantation. Tumor sizes were quantified from 10 representative 12-μm-thick serial coronal brain sections.

### Cell culture

Human glioblastoma cells U251, U87, KNS89, and LN229, as well as HEK-293T cells, were grown in Dulbecco's Modified Eagle Medium (DMEM) (11995040, Gibco, USA) containing 10% fetal bovine serum (FBS) (10099141, Gibco, USA) and 1% penicillin/streptomycin (15140122, Gibco, USA). Glioblastoma cells were cultured in confocal laser scanning microscopy-compatible 24-well plates with ultra-thin bottoms (P24-1.5H-N, Cellvis, USA). Neuronal induction medium consists of DMEM, F12 and neurobasal (2:2:1, 11995040, 11765054, 21103049, Gibco, USA), N2 (17502001, Gibco, USA), B27 (17504044, Gibco, USA), forskolin (10 µM, S2449, Selleck, USA), and dorsomorphin (1 µM, S7840, Selleck, USA) was utilized to cultivate these cells until the end of reprogramming, two days after infection with the virus packed in HEK-293T. To block the action of DAPK1 and RhoA, TC-DAPK6 (HY-15513) and Rhosin hydrochloride (HY-12646), acquired from MCE (USA), were employed.

### Plasmid assembly and lentiviral packaging

In brief, 97mer oligos were manufactured by Sangon Biotech (China) (**Table S1**) and amplified by polymerase chain reaction (PCR), then put into specific lentiviral vectors containing M-cherry marker following digestion with restriction endonuclease (NEB, USA), and then transformed into E. coli for amplification and sequencing 24, 25. M-cherry and HA tagged DAPK1, UNC5B, PTBP1, and PTEN plasmids were provided by Youze Bio (China). The lentiviral vectors and packaging plasmids (pMDL, VSV-G, and pRSV) were then transfected into HEK-293T cells, and the media were replaced after 14 h. The cell supernatant obtained after 24 and 48 h were filtered and used for cell infection. The infection efficiencies of U251, U87, KNS89 and LN229 cells (MOI = 5) are depicted in **Figure S1A, B**. In the unbiased sh-RNA screen, designers constructed lentiviruses (including two sh-RNAs per candidate gene) and numbered them. Unaware operators used these viruses to interfere with *PTBP1* knockdown-induced reprogramming, followed by ICF staining. In addition, images used for analysis were taken at fixed positions of the plate.

### Western blot analysis

RIPA lysis buffer (89900, Thermo Fisher Scientific, USA) was used to extract total proteins from cells and tissues, which were then measured using a bicinchoninic acid (BCA) Protein Assay Kit (23227, Thermo Fisher Scientific, USA). Sodium dodecyl sulfate-polyacrylamide gel electrophoresis separated the protein samples (n = 3), followed by their transfer onto polyvinylidene fluoride membranes. Membranes were imaged by manual exposure technique after 2 h of blocking in 5% milk, 16 h of primary antibody (PTBP1 (DF6644); UNC5B (DF13685); NTN1 (DF8579); DAPK1 (DF8030); Phospho-DAPK1 (Ser308, AF8046); Phospho-P53 (Ser20, AF3073); caspase 3 (p17, AF7022); RGMA (DF13622); HA-tag (T0050); PTEN (AF5447) and GAPDH (AF7021) antibodies from Affinity (USA) and NTN4 (A13775) antibody from ABclonal (China), 1:1000) incubation and 2 h of secondary antibody (S0001, Affinity, USA, 1:5000) incubation. Immunoreactive bands were analyzed using Image J software (v1.8.0).

### Real-time quantitative PCR (qRT-PCR)

TRIzol reagent (15596018, Thermo Fisher Scientific, USA) was used to extract total RNA from cells, and then 2 μg RNA per sample (n = 3) was reverse transcribed using Revert Aid First Strand cDNA Synthesis Kit (K1622, Thermo Fisher Scientific, USA). Primer-BLAST was used to design the primers for all target genes manufactured by Sangon Biotech (China) (**Table S2**). qRT-PCR was conducted using Iraq Universal SYBR Green supermix (1725124, BIO-RAD, USA). The target genes' cycle threshold (Ct) values were normalized to that of the internal control gene (GAPDH), and relative changes in gene expression were computed using the formula 2^-ΔΔCt^.

### Immunofluorescence (IF)

The left ventricle of nude mice was perfused with 20 mL of normal saline and 20 mL of 4% paraformaldehyde successively. Following that, brain tissue was sampled for dehydration, fixation and frozen section. Glioblastoma cells and brain slices were fixed for 20 min in 4% paraformaldehyde (brain slices were then repaired with Quick Antigen Retrieval Solution (P0090, Beyotime, China)), then incubated for 30 min in PBST (0.4% triton in PBS) containing 5% bovine serum albumin (BSA) (ST025, Beyotime, China) solution to inhibit non-specific staining. After that, samples were incubated at 4 °C overnight with primary antibodies (TUJ1 (ab78078), MAP2 (ab96378), KI67 (ab15580) and DCX (ab207175) from Abcam, USA, 1:1000) and then at 37 °C for 1 h with secondary antibodies (ab150077, ab150115 from Abcam, USA, 1:1000). The nuclei were counterstained with DAPI (ab285390, Abcam, USA). ICF and IHF staining were scanned and analyzed using a confocal laser scanning microscopy (OLYMPUS, JPN) and Image J software (v1.8.0). Nine random fields from triplicate cell samples or 45 random fields from a series of every tenth coronal brain section of five nude mice were collected for quantification.

### Cell Proliferation and survival Assays

The proliferation of cells and brain tissues was detected using BeyoClick^TM^ EdU Cell Proliferation Kit with Alexa Fluor 488 (C0071S, Beyotime, China), which is based on incorporating the thymidine analog EdU (5-ethynyl-2'-deoxyuridine) in DNA synthesis process 26. EdU was administered to the cell culture medium (10 μM, 2-h incubation) or infused into the abdominal cavity (50 mg/kg, 4-h therapy) of mice in advance and then tagged with Alexa Fluor 488 via a click reaction. Following reprogramming or treatment with inhibitors, the cell survival rate was determined using Cell Counting Kit-8 (CCK8). The cells in 96-well plate were treated with 10 μL CCK-8 solution, and incubated for 2 h. The absorbance of each well was then quantified at 450 nm.

### PP2A and RhoA activation assays

PP2A activity was determined by the kit purchased from Haling Bio (50042.3 v.A, China) based on the reaction with free phosphate released by the dephosphorylation function of PP2A, and its absorbance can be detected at 660 nm. G-LISA RhoA activation assays (BK124, Cytoskeleton, USA) are ELISA-based RhoA activation assays that can help measure RhoA activity in reprogrammed cells. The level of activation is measured with absorbance set at 490 nm.

### RNA Sequencing (RNA-seq)

The raw data from Illumina HiSeq sequencing was filtered and compared to the reference sequence, which is the foundation of quantitative analysis of known and novel genes. Differentially expressed genes (Fold Change > 2, FDR < 0.05 in sh-Luci-3d vs. sh-PTBP1-3d, FDR < 0.01 in sh-PTBP1-3d vs. sh-PTBP1-7d) between samples (n = 3) were sorted according to P-value and then excavated by STRING (v11.5).

The sequencing data has been submitted to national center for biotechnology information (NCBI) Gene Expression Omnibus (GEO, https://www.ncbi.nlm.nih.gov/geo/info/linking.html.) under the accession number GSE189816. In addition, FPKM value file was provided in the supplementary materials (**Table S3**).

### Statistical Analysis

The statistical analysis was conducted using Graph Pad Prism software (v7.04). The data were presented in the form of means ± standard deviation (SD). The Student's t-test was utilized to determine the difference between the two groups. One-way analysis of variance (ANOVA) was used to compare three or more groups. The log-rank (Mantel-Cox) test was performed to compare Kaplan-Meier survival curves. P < 0.05 was considered statistically significant.

## Results

### PTBP1 knockdown promotes neural differentiation of glioblastoma cells

**Figure 1:**
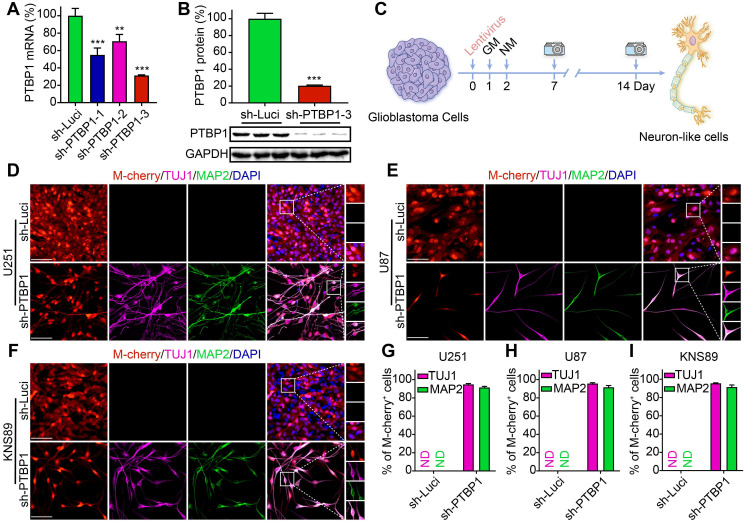
*PTBP1* knockdown reprograms glioblastoma cells into a neural differentiation state. (**A**) Knockdown efficiency of three different sh-PTBP1 lentiviruses detected by qRT-PCR (n = 3). (**B**) Western blot analysis of PTBP1 protein in sh-Luci and sh-PTBP1-3 U251 cells (n = 3). GAPDH was used as an internal reference protein. (**C**) The scheme of cell reprogramming. More than 90% of U251 (**D, G**), U87 (**E, H**), KNS89 (**F, I**) cells expressed neuron markers (TUJ1 and MAP2) at 14 dpi (9 random fields from triplicate samples were captured for quantification; 829 U251, 204 U87 and 540 KNS89 cells (M-cherry+) were tracked per field in sh-Luci group; 164 U251, 20 U87 and 166 KNS89 cells (M-cherry+) were tracked per field in sh-PTBP1 group). The data are presented as mean ± SD. ^**^P < 0.01, ^***^P < 0.001 vs. sh-Luci group. GM: glioblastoma cell medium; ND: not detected; NM: neuronal induction medium. Scale: 100 µm.

To knock down *PTBP1*, three distinct sh-PTBP1 lentiviruses and a control sh-Luci lentivirus were packaged, with M-cherry+ indicating that cells were infected. qRT-PCR and Western blot analyses were utilized to detect changes in PTBP1 mRNA and protein levels in U251 glioblastoma cells three days post infection (dpi), with more than 95% infection efficiency. The sh-PTBP1-3 lentivirus has the most apparent interference impact on PTBP1 (mRNA level to 31.26%, protein level to 20.44%) (**Figure 1A, B**), which was employed in subsequent analyses and referred to as sh-PTBP1.

Human glioblastoma cells U251, U87, KNS89, and LN229 were seeded in 24-well plates (20,000/well) for reprogramming. The neuronal induction medium was employed for further cell culture two days post infection with sh-Luci and sh-PTBP1, which is beneficial to neuronal survival and maturation 10 (**Figure 1C**). Neuronal morphology and the presence of neuronal markers (early marker TUJ1 and mature marker MAP2) were attributed to successful neuronal induction 27, 28. At 14 dpi, 94.75% of infected U251 cells, 95.61% of infected U87 cells, and 95.51% of infected KNS89 cells were reprogrammed into TUJ1+ cells, while 91.41% of infected U251 cells, 91.55% of infected U87 cells, and 91.43% of infected KNS89 cells were reprogrammed into MAP2+ cells (**Figure 1D-I**). These two markers displayed an identical expression pattern, indicating that reprogramming was successful. Interestingly, LN229 cell line did not exhibit this tendency under similar induction conditions (**Figure S1C, D**). The high TUJ1 and MAP2 positivity rates in M-cherry+ cells led us to conclude that knocking down *PTBP1* alone can efficiently convert specific types of glioblastoma cells into a neural differentiation state.

### PTBP1 knockdown suppresses the proliferation rate of glioblastoma cells

**Figure 2:**
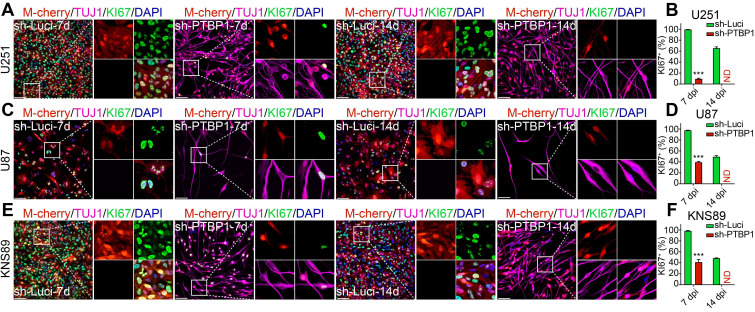
*PTBP1* knockdown-mediated reprogramming silences the proliferative marker KI67 in glioblastoma cells. Immunocytofluorescent analysis of U251 (**A-B**), U87 (**C-D**), and KNS89 (**E-F**) cell proliferation at 7 and 14 dpi using KI67 detection (9 random fields from triplicate samples were captured for quantification; KI67^+^ (%) = KI67^+^ M-cherry^+^/M-cherry^+^; 546-851 U251, 114-205 U87 and 421-692 KNS89 cells (M-cherry+) were tracked per field in sh-Luci group; 159-172 U251, 17-21 U87 and 122-167 KNS89 cells (M-cherry+) were tracked per field in sh-PTBP1 group)**.** The data are presented as mean ± SD. *** P < 0.001 vs. sh-Luci group. Dpi (d): days post infection; ND: not detected. Scale: 100 µm.

**Figure 3:**
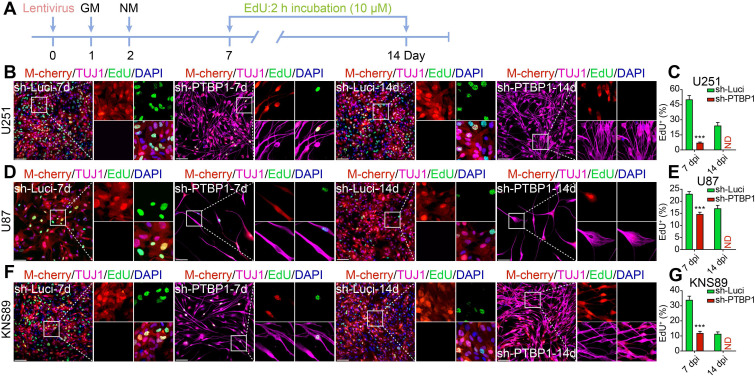
*PTBP1* knockdown suppresses the proliferative marker EdU in glioblastoma cells. **(A)** The experimental design for labeling EdU in glioblastoma cells. EdU detection of U251 (**B-C**), U87 (**D-E**), and KNS89 (**F-G**) cell proliferation at 7 and 14 dpi (9 random fields from triplicate samples were captured for quantification; EdU^+^ (%) = EdU^+^ M-cherry^+^/M-cherry^+^; 565-831 U251, 121-211 U87 and 440-684 KNS89 cells (M-cherry+) were tracked per field in sh-Luci group; 161-185 U251, 20-24 U87 and 130-159 KNS89 cells (M-cherry+) were tracked per field in sh-PTBP1 group). The data are presented as mean ± SD. ***P < 0.001 vs. sh-Luci group. Dpi (d): days post infection; GM: glioblastoma cell medium; ND: not detected; NM: neuronal induction medium. Scale: 100 µm.

Inhibiting cancer cell proliferation to enhance patient health outcomes is research priority for all malignancies. KI67 protein and EdU were chosen to examine the ability of reprogrammed glioblastoma cells to proliferate. The high expression of KI67 protein in cycling cells and the considerable decrease in resting G0 cells are used to assess cancer cell proliferation potential, indicating a malignant degree 29. At 7 dpi, KI67+ rates of infected U251, U87, and KNS89 glioblastoma cells decreased from 99.47%, 97.48%, and 98.37% to 9.32%, 39.29%, and 41.31%, respectively (**Figure 2**). EdU, a thymidine analog, can be incorporated into newly synthesized DNA during cell proliferation 26. The cells in replicative/S-phase were shown after a 2-h EdU incubation (10 µM) followed by a click reaction (**Figure 3A**). At 7 dpi, EdU+ rates of infected U251, U87, and KNS89 glioblastoma cells decreased from 50.30%, 23.11%, and 33.94% to 7.14%, 14.71% and 11.92%, respectively (**Figure 3B-G**). Even more astonishing, at 14 dpi, KI67 protein and EdU were no longer visible on these infected cells. In this timepoint, not all infected cells were successfully reprogrammed, but they all expressed TUJ1 and MAP2 (some of these cells were not considered to be reprogrammed successfully due to the lack of neuron-like cell morphology). These unreprogrammed cells may be in the early stages of reprogramming or on the verge of cell death, but the proliferation of cells has actually been inhibited 30. This resulted in the phenomenon that neither KI67 nor EdU was expressed in M-cherry+ cells when only about 90% of infected cells were successfully reprogrammed.

The presence of neuronal markers and the absence of proliferation signs indicated that glioblastoma cells U251, U87, and KNS89 were reprogrammed. Similarly, the proliferation rate of infected LN229 glioblastoma cells did not alter significantly, demonstrating that *PTBP1* knockdown cannot affect the fate of all glioblastoma cell lines (**Figure S1E-H**).

### PTBP1 knockdown inhibits the growth of human-derived glioblastoma xenograft *in vivo* through reprogramming

**Figure 4:**
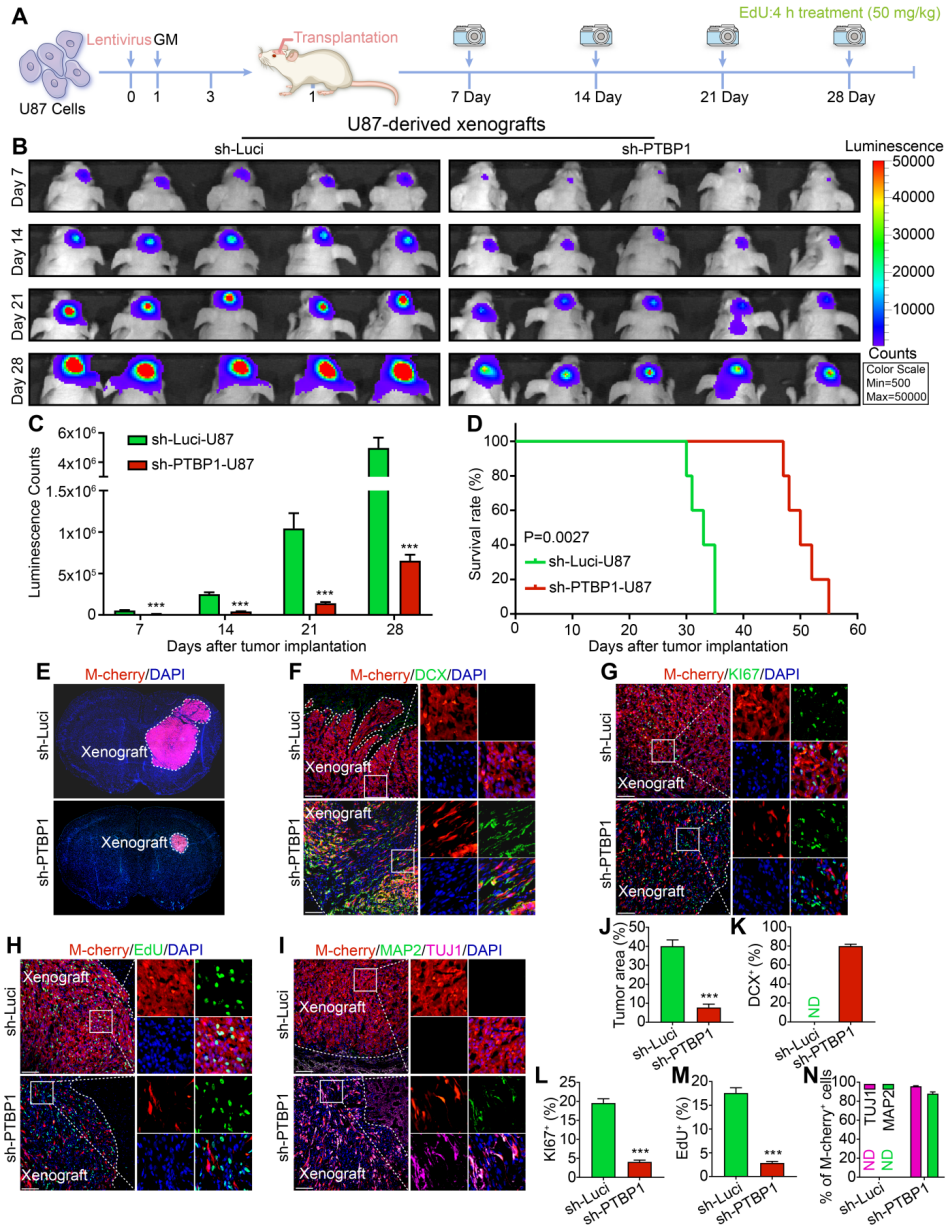
The xenografts derived from *PTBP1* knockdown U87 cells shrink significantly. (**A**) The experimental protocol for orthotopic cell transplantation and EdU pretreatment prior to sacrifice. (**B-C**) *In vivo* bioluminescent images and the quantification of U87-derived xenografts (n = 5). (**D**) Post-imaging Kaplan-Meier survival analysis of transplanted mice (n = 5, P = 0.0027 using log-rank test). (**E, J**) Immunohistofluorescent analysis of tumor formation in mice implanted with sh-Luci and sh-PTBP1 infected U87 cells (n = 5). Tumor mass (outlined by dashed lines) was quantified based on the area occupying ipsilateral brain. (**F, K**) 79.87% of U87 cells expressed immature neuronal marker DCX 28 days after implantation (DCX^+^ (%) = DCX^+^ M-cherry^+^/M-cherry^+^; 1,458 M-cherry+ cells were tracked per field in sh-Luci group; 713 M-cherry+ cells were tracked per field in sh-PTBP1 group). (**G, L**) Lack of KI67 marker of *PTBP1* knockdown U87 cells in xenografts (KI67^+^ (%) = KI67^+^ M-cherry^+^/M-cherry^+^; 1,533 M-cherry+ cells were tracked per field in sh-Luci group; 693 M-cherry+ cells were tracked per field in sh-PTBP1 group). (**H, M**) Lack of EdU marker of *PTBP1* knockdown U87 cells in xenografts (EdU^+^ (%) = EdU^+^ M-cherry^+^/M-cherry^+^; 1,597 M-cherry+ cells were tracked per field in sh-Luci group; 322 M-cherry+ cells were tracked per field in sh-PTBP1 group). (**I, N**) 95.71% and 87.91% of U87 cells expressed neuronal marker TUJ1 and MAP2 42 days after implantation (1,586 M-cherry+ cells were tracked per field in sh-Luci group; 355 M-cherry+ cells were tracked per field in sh-PTBP1 group). Forty-five random fields from a series of every tenth coronal brain section of five nude mice were collected for quantification in immunohistofluorescent analysis. The data are presented as mean ± SD. ***P < 0.001 vs. sh-Luci group. GM: glioblastoma cell medium; ND: not detected. Scale: 100 µm.

We found that knocking down *PTBP1* reprogramed glioblastoma cells like U251, U87, and KNS89 into a neural differentiation state, significantly slowing down their proliferation rate. To investigate its effect *in vivo*, U87 cells, prone to producing masses in the brain, were chosen. A luciferase reporter gene was inserted for orthotopic cell transplantation experiments, as depicted in **Figure 4A**. Sh-Luci-U87 or sh-PTBP1-U87 (3 dpi) were transplanted into the striatum of nude mice, then monitored every seven days with PerkinElmer IVIS Lumina X5 *in vivo* imaging system, and survival time was recorded every day (**Figure 4B-D**). According to findings, the growth of sh-PTBP1-U87 tumor mass in the brain was dramatically reduced, and the survival time of nude mice was significantly increased to 50 days (n = 5, p = 0.0027). Furthermore, the tumor size of sh-PTBP1-U87 in the ipsilateral brain decreased from 40.05% to 7.75% (n = 5, **Figure 4E, J**).

To further evaluate the reprogramming of sh-PTBP1-U87 in nude mice, we performed DCX staining on xenografts 28 days after implantation and found that 79.87% of M-cherry+ cells were successfully expressed this early immature neuronal marker, and the morphology of these M-cherry+ cells was radically different from that of normal U87 cells (**Figure 4F, K**). KI67 and EdU staining also indicated that the growth rate of sh-PTBP1-U87 was dramatically reduced *in vivo* (**Figure 4G, H, L, M**). At 42 days after implantation, 95.71% and 87.91% of sh-PTBP1-U87 expressed the early neuronal marker TUJ1 and the mature neuronal marker MAP2 (**Figure 4I, N**).

In addition, even though U87 cells have an infection efficiency of more than 97% *in vitro*, the proliferation rate of uninfected cells in the brain was substantially higher than that of sh-PTBP1 infected cells, resulting in a sharp decline of M-cherry+ U87 cells *in vivo* to 48.38%. These performances demonstrated that sh-PTBP1-U87 can be reprogrammed in the brain of nude mice.

### RNA-seq detects the changes in the transcriptome after knocking down PTBP1

To further explore the mechanism of *PTBP1* knockdown to initiate reprogramming of glioblastoma cells, we isolated total RNA from sh-Luci infected U251 cells three days post infection (sh-Luci-3d) and sh-PTBP1 infected U251 cells three and seven days post infection (sh-PTBP1-3, 7d) for sequencing.

**Figure 5:**
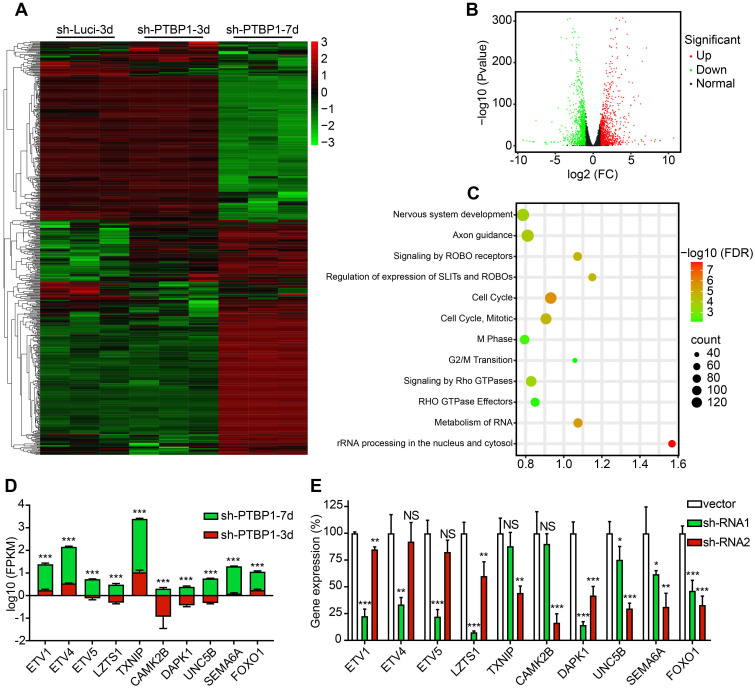
Analysis of RNA sequencing. (**A**) A heatmap shows the expression of 577 distinct genes in sh-Luci-3d, sh-PTBP1-3d, and sh-PTBP1-7d U251 cells (n = 3). The volcano map and pathway enrichment analysis of distinct expression genes between sh-PTBP1-3d and sh-PTBP1-7d U251 cells are shown in **B** and **C**. (**D**) The log10 (FPKM) value of ten candidate genes' roles in glioblastoma cell reprogramming (n = 3). (**E**) To knock down these ten candidates, two sh-RNAs were generated for each gene and validated using qRT-PCR (n = 3). The data are presented as mean ± SD. *P < 0.05, **P < 0.01, ***P < 0.001 vs. sh-PTBP1-3d or sh-Luci group. Dpi (d): days post infection; NS: no significance.

There are 177 different genes between sh-Luci-3d and sh-PTBP1-3d, among which 103 are upregulated and 74 are downregulated, and there are 2269 different genes between sh-PTBP1-3d and sh-PTBP1-7d, among which 1211 genes are upregulated and 1058 are downregulated. The log10 (FPKM) values of 177 genes with significant differences (Fold Change > 2) between sh-Luci-3d and sh-PTBP1-3d, as well as the top 400 genes with significant differences (Fold Change > 2) between sh-PTBP1-3d and sh-PTBP1-7d, were extracted and plotted in a heatmap (**Figure 5A**). As demonstrated, *PTBP1* knockdown did not result in significant variations in the cell transcriptome at three dpi, but numerous genes were altered as the induction proceeded. Therefore, we created a volcano map using the changes in the transcriptome of sh-PTBP1-3d and sh-PTBP1-7d, and used Reactome (v78) to perform pathway enrichment analysis to identify the critical genes (**Figure 5B, C**). The findings indicated that many differentially expressed genes were enriched in nerve growth, cell cycle, and RNA metabolism pathways, consistent with the phenomenon of glioblastoma cells transforming into neural differentiation cells. We screened ten candidates (**Figure 5D**) based on enrichment analysis results and the significance of differential gene expressions that may play an essential role in the reprogramming process caused by *PTBP1* knockdown due to their specific functions. This includes ETS variant transcription factor family (*ETV1*, *ETV4*, and *ETV5*) involved in neuronal differentiation 31; Leucine zipper tumor suppressor 1 (*LZTS1*), calcium/calmodulin-dependent protein kinase II beta (*CAMK2B*), unc-5 netrin receptor B (*UNC5B*) and semaphorin 6A (*SEMA6A*) for neuronal development and axon branching 32-35; Death associated protein kinase 1 (*DAPK1*) involved in neuronal survival 36; Thioredoxin interacting protein (*TXNIP*) for cell cycle control and forkhead box O1 (*FOXO1*) for cell transformation 37, 38. In addition, sh-RNA lentiviruses were created to knock down these target genes for future research (**Figure 5E**).

### UNC5B receptor is involved in the reprogramming caused by PTBP1 knock down

TUJ1, an early neuronal marker, was already expressed in reprogrammed cells at 7 dpi, while MAP2 was not expressed until day 14. In the screening process, we selected TUJ1 and KI67 as indicators to detect the reprogramming state of U251 cells after 7 days of specific induction. We discovered that sh-DAPK1 and sh-UNC5B effectively reversed the reprogramming of U251 cells mediated by sh-PTBP1.

**Figure 6:**
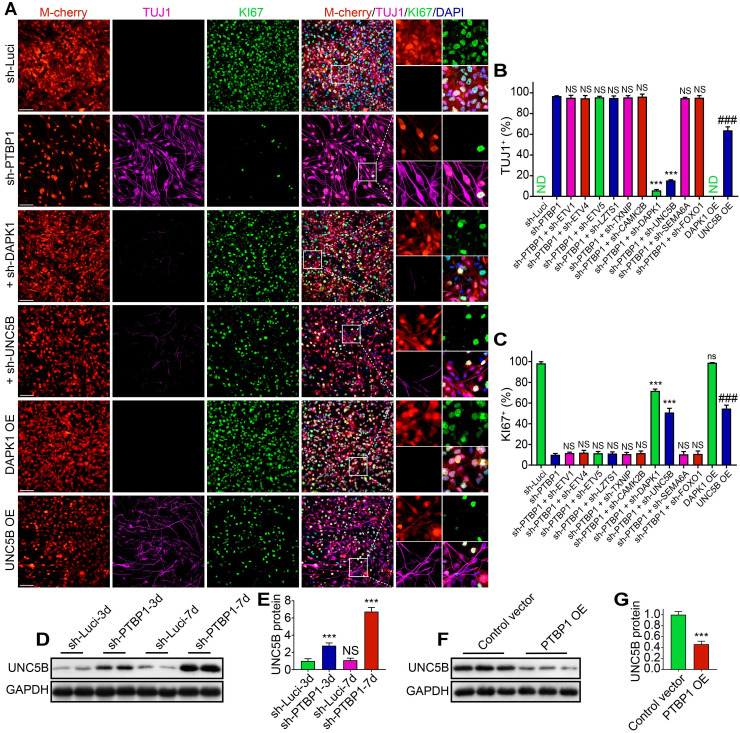
*UNC5B* receptor is a key in reprogramming generated by *PTBP1* knockdown. (**A-C**) KI67 and TUJ1 staining revealed the reprogramming efficiency of particular lentiviruses-infected U251 cells (9 random fields from triplicate samples were captured for quantification; TUJ1^+^ (%) = TUJ1^+^ M-cherry^+^/M-cherry^+^; KI67^+^ (%) = KI67^+^ M-cherry^+^/M-cherry^+^; M-cherry+ cells = 128-566 for each condition). (**D, E**) Western blot analysis of UNC5B protein in U251 cells infected with sh-Luci and sh-PTBP1 for 3 and 7 days. (n = 3). (**F, G**) The change of UNC5B protein expression under the state of *PTBP1* overexpression. (n = 3). GAPDH was used as an internal reference protein. The data are presented as mean ± SD. ***P < 0.001, ###P < 0.001 vs. sh-PTBP1, sh-Luci, sh-Luci-3d or control vector group. Dpi (d): days post infection; ND: not detected; NS (ns): no significance; OE: overexpression. Scale: 100 µm.

At 7 dpi, TUJ1+ rate of sh-PTBP1 + sh-DAPK1 group and sh-PTBP1 + sh-UNC5B group declined from 96.95% to 5.79% and 15.44%, respectively, whereas KI67 increased from 10.05% to 71.87% and 50.92%, respectively, compared to sh-PTBP1 group (**Figure 6A-C**). UNC5B belongs to UNC5 axon guidance gene family and is a receptor for netrins (NTN), which functions in neuron growth 39. DAPK1, a calcium/calmodulin-regulated protein kinase that activates death signaling pathway, is a downstream of UNC5B 40. We then placed *DAPK1* and *UNC5B* overexpressed U251 cells in the reprogramming environment and found that *UNC5B* overexpression could induce 63.75% of infected U251 cells to express TUJ1, and KI67 rate was also decreased to 54.74%, while *DAPK1* overexpression had no effect on U251 differentiation (**Figures 6A-C and S2A, B**). Western blot analyses revealed that protein levels of UNC5B in sh-Luci and sh-PTBP1 increased 2.78-fold and 6.74-fold at 3 dpi and 7 dpi, respectively (**Figure 6D, E**). Moreover, UNC5B protein expression was affected by *PTBP1* overexpression (**Figures 6F, G and S2C**). Based on the above experiments, we believe that UNC5B is a critical node in reprogramming induced by *PTBP1* knockdown, but this is not the whole reason, since *UNC5B* overexpression cannot fully replicate the effects induced by *PTBP1* knockdown.

### DAPK1 inhibitor prevents cell reprogramming caused by PTBP1 knockdown

**Figure 7:**
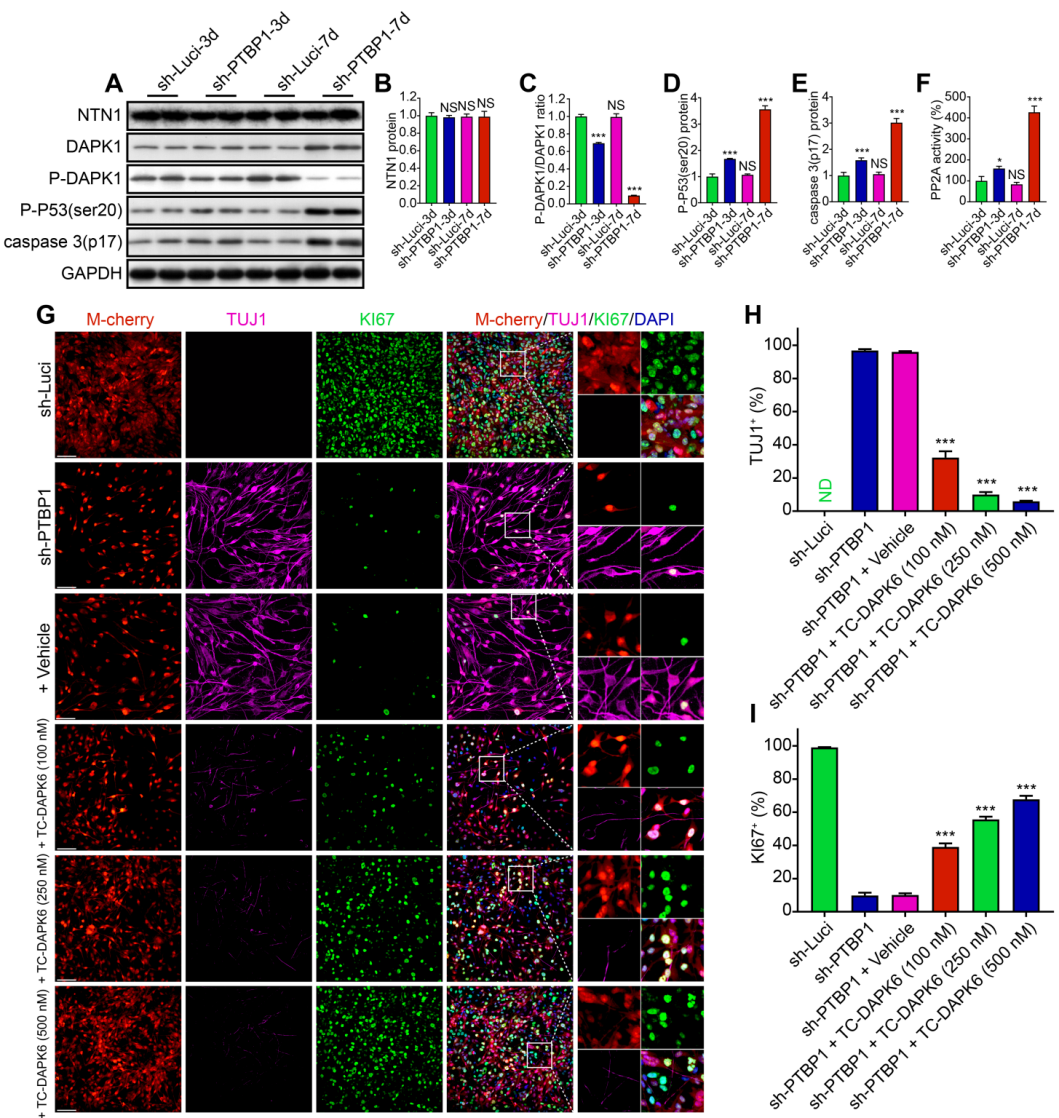
A DAPK1 inhibitor prevents the *PTBP1* knockdown induced reprogramming. (**A-E**) Western blot study of NTN1, DAPK1, P-DAPK1(Ser308), P-P53(ser20), and caspase 3(p17) protein in U251 cells infected with sh-Luci and sh-PTBP1 for 3 and 7 days. (n = 3). As an internal reference protein, GAPDH was used. (**F**) Activity of PP2A during reprogramming. (n = 3). (**G-I**) TUJ1 and KI67 positive rates of six distinct groups constituted of lentivirus (sh-Luci or sh-PTBP1) and TC-DAPK6 (vehicle, 100 nM, 250 nM, and 500 nM; nine random fields from triplicate samples were recorded for quantification; TUJ1^+^ (%) = TUJ1^+^ M-cherry^+^/M-cherry^+^; KI67^+^ (%) = KI67^+^ M-cherry^+^/M-cherry^+^; M-cherry+ cells = 156-553 for each condition). The data are presented as mean ± SD. *P < 0.05, *** P < 0.001 vs. sh-Luci-3d or sh-PTBP1 + vehicle group. Dpi (d): days post infection; ND: not detected; NS: no significance. Scale: 100 µm.

Following the prior findings, UNC5B-DAPK1 pathway may play a significant role in PTBP1 knockdown-induced cell reprogramming. Therefore, we isolated sh-Luci-3, 7d and sh-PTBP1-3, 7d proteins from U251 cells for Western Blot analysis. As displayed in **Figure 7A-E**, there was no significant change in NTN1 protein expression, resulting in over-expressed UNC5B receptors with insufficient NTN1 to bind to it. These unloaded receptors dephosphorylated DAPK1 proteins and activated PP2A to form a protein complex 40 (**Figure 7F**), and then induced phosphorylation of the ser20 site of P53 protein and promoted caspase 3 protein activation 41. Western blot analysis results are compatible with transcriptome sequencing.

To further validate the role of UNC5B-DAPK1 pathway in *PTBP1* knockdown and the resulting reprogramming of glioblastoma cells, subsequent experiments were conducted for blocking tests and six groups were created: sh-Luci, sh-PTBP1, sh-PTBP1 + vehicle, and sh-PTBP1 + varying concentrations of TC-DAPK 6 (100 nM, 250 nM, and 500 nM). TC-DAPK 6 is a DAPK1 inhibitor that can reduce DAPK1 activity by 50% at 69 nM 42 and has been shown to have no effect on the proliferation of U251 cells at concentrations less than 1000 nM (**Figure S2D**). It was revealed that TC-DAPK 6 could significantly inhibit *PTBP1* knockdown mediated reprogramming. At 7 dpi, the positive rate of TUJ1 reduced from 32.27% (100 nM treatment) to 5.90% (500 nM treatment), while the positive rate of KI67 increased from 39.05% (100 nM treatment) to 67.79% (500 nM treatment) (**Figure 7G-I**). These findings are consistent with the behavior of sh-PTBP1 + sh-DAPK1 infected U251 cells. In general, sh-DAPK1 and TC-DAPK 6 suppress DAPK1 catalytic activity, allowing U251 glioblastoma cells to proliferate and then disrupt the reprogramming process.

### RhoA inhibitor prevents cell reprogramming caused by PTBP1 knockdown

**Figure 8:**
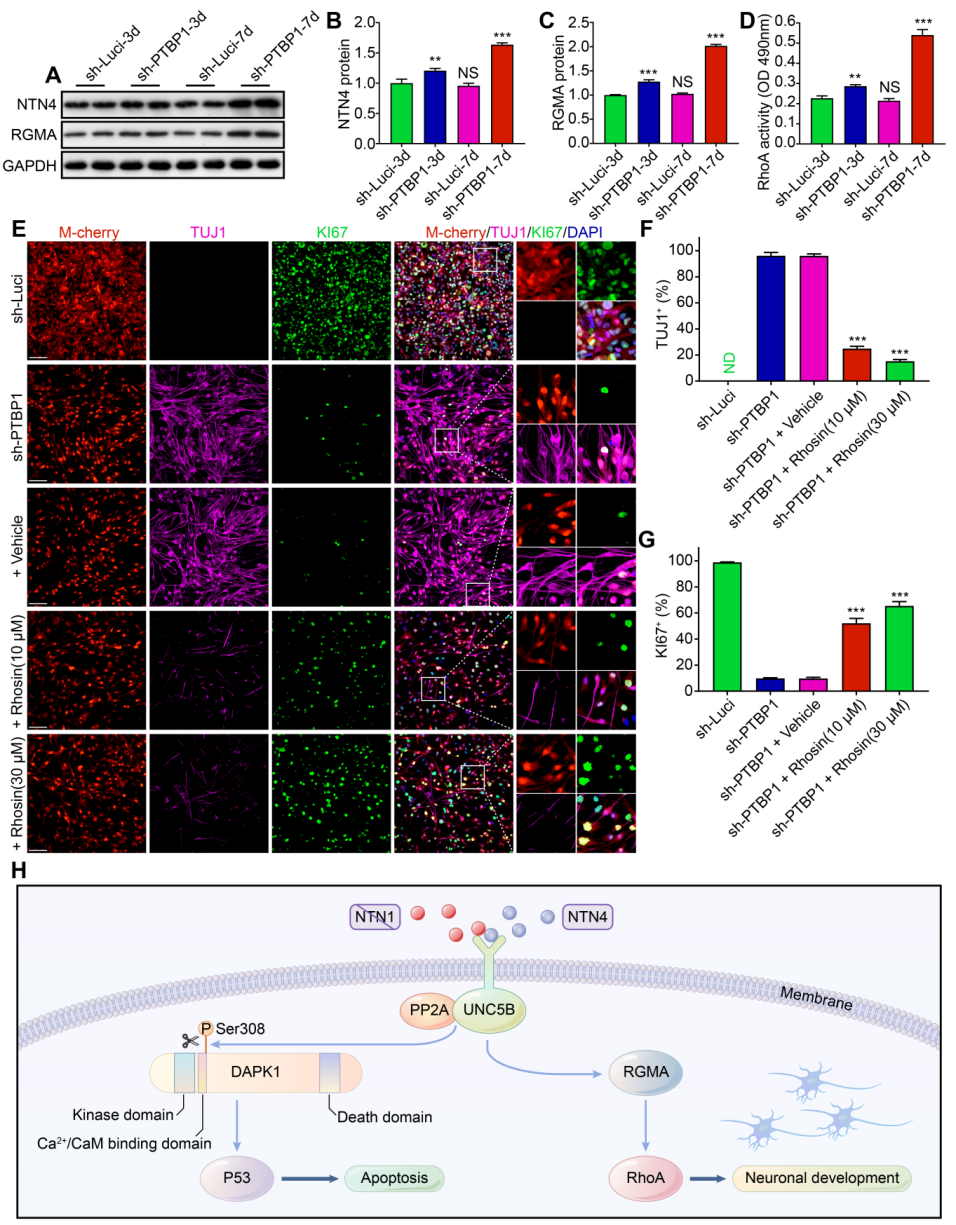
A RhoA inhibitor prevents the *PTBP1* knockdown induced reprogramming. (**A-C**) Western blot study of NTN4 and RGMA protein in U251 cells infected with sh-Luci and sh-PTBP1 for 3 and 7 days. (n = 3). GAPDH was used as an internal reference protein. (**D**) RhoA activity measured by G-LISA. (n = 3). (**E-G**) TUJ1 and KI67 positive rates of five distinct groups constituted of lentivirus (sh-Luci or sh-PTBP1) and Rhosin (vehicle, 10 µM, and 30 µM; nine random fields from triplicate samples were recorded for quantification; TUJ1^+^ (%) = TUJ1^+^ M-cherry^+^/M-cherry^+^; KI67^+^ (%) = KI67^+^ M-cherry^+^/M-cherry^+^; M-cherry+ cells = 286-538 for each condition). (**H**) Pathways involved in *PTBP1* knockdown-induced reprogramming. The data are presented as mean ± SD. **P < 0.01, *** P < 0.001 vs. sh-Luci-3d or sh-PTBP1 + vehicle group. Dpi (d): days post infection; ND: not detected; NS: no significance. Scale: 100 µm.

Through RNA-seq data mining and western blot verification, we found that the expression of NTN4 protein (UNC5B upstream) and RGMA protein (UNC5B downstream) also changed in sh-PTBP1 infected U251 cells and increased with induction time prolongation 34, 43 (**Figure 8A-C**). Similar changes were detected in the activity of RhoA, which is a downstream of RGMA protein (**Figure 8D**). Moreover, NTN4-UNC5B pathway activation was demonstrated to promote axonal development in a previous study 43.

To verify the role of this pathway in *PTBP1* knockdown-induced reprogramming, we designed a blocking test with RhoA inhibitor (Rhosin (EC50: 10-30 μM)), which had no effect on U251 proliferation at concentrations below 50 μM 44 (**Figure S2E**). It was revealed that Rhosin could significantly inhibit *PTBP1* knockdown-mediated reprogramming. At 7 dpi, the positive rate of TUJ1 reduced from 96.10% (sh-PTBP1 + vehicle) to 15.23% (sh-PTBP1 + 30 μM treatment), while the positive rate of KI67 increased from 9.69% (sh-PTBP1 + vehicle) to 65.41% (sh-PTBP1 + 30 μM treatment) (**Figure 8E-G**).

Based on the above experiments, we believe that glioblastoma cells were reprogrammed to a neural differentiation phase in a dynamic balance of UNC5B receptor-induced cell death via DAPK1-P53 pathway and promotion of axonal growth via RGMA-RhoA pathway 45, 46 (**Figure 8H**).

## Discussion

We successfully reprogrammed three glioblastoma cell lines, U251, U87, and KNS89, into a neural differentiation state by knocking down *PTBP1*. The reprogramming efficiency has reached more than 90%, which is a significant improvement over 20-40% of Ascl1, Brn2, and Ngn2 overexpression 47, suggesting that *PTBP1* knockdown has met our criteria for modifying cancer progression. The cell survival assay revealed that growth of *PTBP1* knockdown U251, U87 and KNS89 cells was significantly inhibited in the reprogramming environment than in the normal environment, while reprogramming environment had limited proliferation inhibition on sh-Luci infected cells (**Figure S2F**). Therefore, we believe that neuronal reprogramming caused by *PTBP1* knockdown greatly inhibited the proliferation of glioblastoma cells, not just the effect of *PTBP1* knockdown itself. Additionally, tumor-bearing studies demonstrated that *PTBP1* knockdown could reduce tumor volume by fivefold after 28 days and increase the survival duration of transplanted mice compared to the control group. sh-PTBP1-U87 was also demonstrated to be reprogrammed successfully in the brain of nude mice.

The sequencing and *in vitro* studies indicated that UNC5B receptor was involved in *PTBP1* knockdown induced reprogramming. In addition, *UNC5B* overexpression alone can induce a part of U251 cells to express neuronal marker. UNC5B was reported to involve in neuronal growth as an NTN-binding receptor and perform a dual role in apoptosis 39, 40, 43. Under normal conditions, nutrient ligands bind to UNC5B to inhibit apoptosis. However, when NTN1 is missing, UNC5B binds PP2A and DAPK1 to form a complex and dephosphorylates the ser308 site of DAPK1, enhancing its catalytic activity and boosting apoptosis. Western blot analyses of these proteins extracted from xenografts demonstrated changes consistent with *in vitro* study (**Figure S2G-O**). Based on the above evidences, UNC5B receptor is believed to reprogram glioblastoma cells under the function of enhancing neuron development caused by adequate NTN4 binding and promoting apoptosis by inadequate NTN1 binding. Such promotion of cell death in neuronal reprogramming has also been reported by Gascon and his colleagues 30. In the infected cells, they found that 94% of those without neuronal morphology underwent cell death, compared with 48% of those with neuronal morphology at the time of cell fate conversion.

A DAPK1 inhibitor partially prevented the reprogramming of glioblastoma cells produced by *PTBP1* knockdown, indicating the participation of UNC5B-DAPK1 pathway once again. DAPK1 regulates programmed cell death via P53 as a calcium/calmodulin (Ca^2+^/CaM) regulated serine/threonine kinase 41. *DAPK1* expression in glioblastomas has been demonstrated to be decreased 48, and glioma patients with high *DAPK1* expression had a longer survival time according to TCGA (**Figure S2P**). Furthermore, reduced *DAPK1* expression has been documented in renal, colorectum, and liver cancers 49-51. These findings suggest that DAPK1 repression is likely to cause cancer cell proliferation, consistent with the phenomena of enhanced DAPK1 activity and slower glioblastoma cell proliferation produced by *PTBP1* knockdown in this work. As a result, DAPK1 may be a potential target for glioblastoma therapy and warrants further investigation. Besides, RhoA inhibitor could also block PTBP1 knockdown-induced reprogramming. Although it is controversial whether RhoA activation promotes or inhibits neuronal cell growth in existing reports 52, RhoA activation was involved in *PTBP1* knockdown-induced reprogramming in this study.

Unfortunately, knocking down *PTBP1* does not allow all types of glioblastoma cells to enter a neural differentiation stage. We detected PTBP1 protein expression in four glioblastoma cell lines and found that they were all PTBP1-abundant, with being slightly higher in U251 and KNS89 cells (**Figure S2Q, R**). This indicates that differential PTBP1 expression is not the key to determine whether *PTBP1* knockdown can reprogram glioblastoma cells. Based on previous studies and comparisons between cell lines, PTEN protein was absent in these three cell lines that could be successfully reprogrammed due to mutations, whereas LN229 was normally expressed 53 (**Figure S2S**). In addition, PTEN was involved in the differentiation process of various cells 54, 55. Therefore, we speculate that PTEN protein may be the switch to determine whether the cell line can be reprogrammed. We then overexpressed PTEN protein in U251, U87 and KNS89 cells and found that they were no longer affected by *PTBP1* knockdown (**Figure S2T-W**). Furthermore, the safety of cell reprogramming mediated by *PTBP1* knockdown should be investigated in the future.

## Supplementary Material

Supplementary figures and tables 1-2.

Supplementary table 3.

## Abbreviations

ANOVA: analysis of variance
BCA: bicinchoninic acid
CAMK2B: calcium/calmodulin dependent protein kinase II beta
DAPK1: death associated protein kinase 1
DMEM: Dulbecco's Modified Eagle Medium
dpi: days post infection
EdU: 5-ethynyl-2'-deoxyuridine
ETV: ETS variant transcription factor
FBS: fetal bovine serum
FOXO1: forkhead box O1
GEO: Gene Expression Omnibus
GM: glioblastoma cell medium
ICF: immunocytofluorescence
IDH: isocitrate dehydrogenase
IHF: immunohistofluorescence
IF: immunofluorescence
LZTS1: leucine zipper tumor suppressor 1
NCBI: national center for biotechnology information
ND: not detected
NM: neuronal induction medium
NS: no significance
NTN: netrins
OE: overexpression
OS: overall survival time
PCR: polymerase chain reaction
PP2A: protein phosphatase 2A
PTBP1: polypyrimidine tract binding protein 1
qRT-PCR: real-time quantitative PCR
SD: standard deviation
SEMA6A: semaphorin 6A
TCGA: The Cancer Genome Atlas
TXNIP: thioredoxin interacting protein
UNC5B: unc-5 netrin receptor B
WHO: World Health Organization
