# Supplementary material for: PTBP1 knockdown promotes neural differentiation of glioblastoma cells through UNC5B receptor
Source: Theranostics. 2022 May 9;12(8):3847–61. doi: 10.7150/thno.71100 (PMC9131277; doi:10.7150/thno.71100)
Supplement: Supplementary file 1 — Supplementary figures and tables 1-2. [file thnov12p3847s1.pdf]

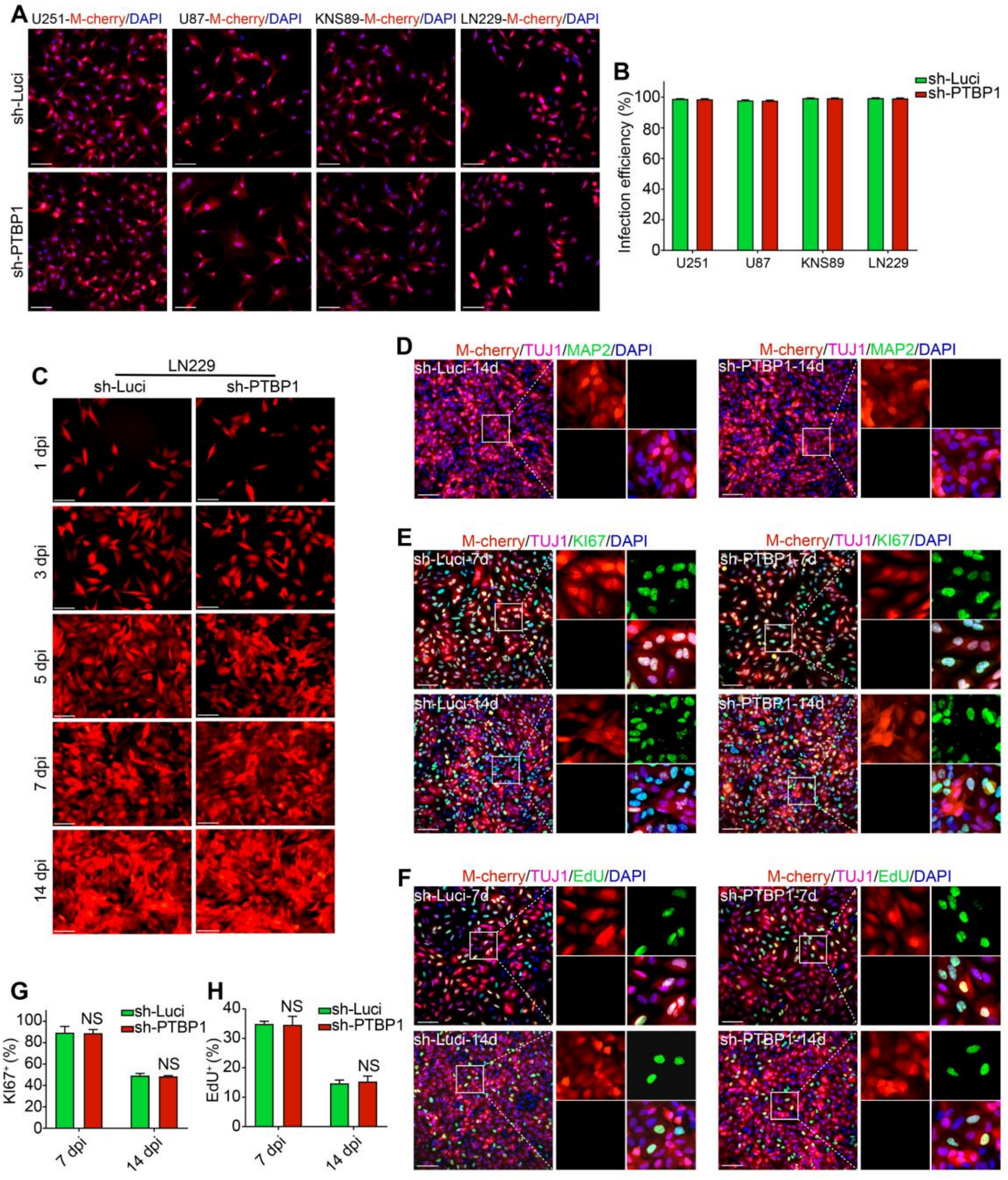

Figure S1. (**A, B**) Infection efficiency of sh-Luci and sh-PTBP1 in U251, U87, KNS89 and LN229 cells at 3 dpi (47-109 cells were tracked per field in sh-Luci group; 40-105 cells were tracked per field in sh-PTBP1 group). (**C**) Under low PTBP1 protein expression, LN229 cells showed no significant morphological alterations. (**D**) Neuronal makers (TUJ1 and MAP2) cannot be detected in LN229 cells infected with sh-Luci or sh-PTBP1. (**E-H**) Immunocytofluorescent analysis of LN229 cell proliferation using

KI67 and EdU detection at 7 and 14 dpi. In immunocytofluorescent analysis, nine random fields from triplicate samples were measured for quantification ($KI67^+$ (%) = $KI67^+$ M-$cherry^+$/M-$cherry^+$; EdU^+ (%) = EdU^+ M-$cherry^+$/M-$cherry^+$; 321-869 M-cherry+ LN229 cells were tracked per field in sh-Luci group; 339-862 M-cherry+ LN229 cells were tracked per field in sh-PTBP1 group). The data are presented as mean ± SD. No significance vs. sh-Luci group. Dpi (d): days post infection; NS: no significance. Scale: 100 μm.

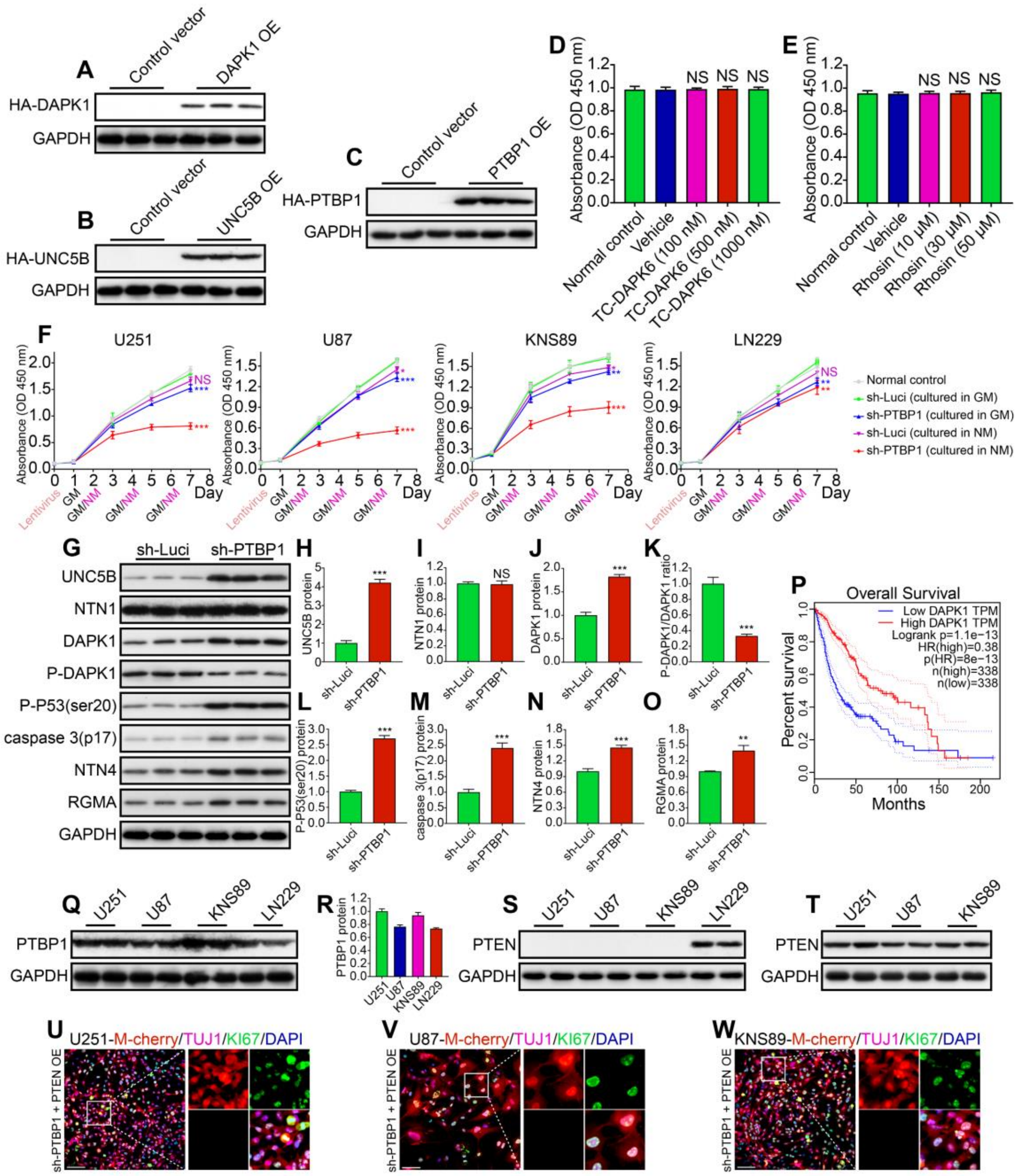

Figure S2. (**A-C**) Western blot analysis of HA-DAPK1, HA-UNC5B and HA-PTBP1 protein in U251 cells infected with overexpression lentiviruses for three days. (n = 3). As an internal reference protein, GAPDH was used. (**D, E**) CCK8 assays under different concentrations of TC-DAPK6 or Rhosin. (n = 3). (**F**) Except for LN229, the growth of reprogrammed U251, U87 and KNS89 cells was significantly inhibited. (n = 3). (**G-O**) Western blot analysis of UNC5B, NTN1, DAPK1, P-DAPK1(ser308), P-P53(ser20), caspase 3(p17), NTN4 and RGMA in xenografts derived from sh-Luci and sh-PTBP1

infected U87 (n = 3). As an internal reference protein, GAPDH was used. (**P**) Survival analysis of DAPK1 in glioma patients (based on TCGA data in GEPIA). (**Q-S**) Western blot analysis of PTBP1 and PTEN proteins in normal U251, U87, KNS89 and LN229 cells. (n = 3). GAPDH was used as an internal reference protein. (**T**) PTEN protein was overexpressed in U251 U87 and KNS89 cells. GAPDH was employed as an internal reference protein. (**U-W**) *PTBP1* knockdown induced reprogramming was blocked by normal PTEN protein in U251, U87 and KNS89 cells. The data are presented as mean ± SD. *P < 0.05, **P < 0.01, ***P < 0.001 vs. vehicle or sh-Luci group. GM: glioblastoma cell medium; NM: neuronal induction medium; NS: no significance; OE: overexpression. Scale: 100 μm.

Table S1. List of the sh-RNA sequences.

Identifier	Sequence（5'to3'）
sh-PTBP1-1	TGCTGTTGACAGTGAGCGCTAGCAAGATGATACAATGGTATAGTGAAGCCACAGATGTATACCATTGTATCATCT TGCTATTGCCTACTGCCTCGGA
sh-PTBP1-2	TGCTGTTGACAGTGAGCGCGCGCGTGAAGATCCTGTTCAATAGTGAAGCCACAGATGTATTGAACAGGATCTTCA CGCGCTTGCCTACTGCCTCGGA
sh-PTBP1-3	TGCTGTTGACAGTGAGCGCAGGATTCAAGTTCTTCCAGAATAGTGAAGCCACAGATGTATTCTGGAAGAACTTGA ATCCTTTGCCTACTGCCTCGGA
ETV1 sh-RNA1	TGCTGTTGACAGTGAGCGCAGGGTTGAAAATATTACATTATAGTGAAGCCACAGATGTATAATGTAATATTTTCA ACCCTTTGCCTACTGCCTCGGA
ETV1 sh-RNA2	TGCTGTTGACAGTGAGCGCCCCAGTTTATTCAGAGCTCAATAGTGAAGCCACAGATGTATTGAGCTCTGAATAAA CTGGGTTGCCTACTGCCTCGGA
ETV4 sh-RNA1	TGCTGTTGACAGTGAGCGACCCTGTGTACATATAAATGAATAGTGAAGCCACAGATGTATTCATTTATATGTACA CAGGGCTGCCTACTGCCTCGGA
ETV4 sh-RNA2	TGCTGTTGACAGTGAGCGACCGCTCGCTCCGATACTATTATAGTGAAGCCACAGATGTATAATAGTATCGGAGCG AGCGGCTGCCTACTGCCTCGGA
ETV5 sh-RNA1	TGCTGTTGACAGTGAGCGCCAGGATCTCAGTCAACTTCAATAGTGAAGCCACAGATGTATTGAAGTTGACTGAGA TCCTGATGCCTACTGCCTCGGA
ETV5 sh-RNA2	TGCTGTTGACAGTGAGCGCTAGCATTGTACTCTAATCAAATAGTGAAGCCACAGATGTATTTGATTAGAGTACAA TGCTAATGCCTACTGCCTCGGA
LZTS1 sh-RNA1	TGCTGTTGACAGTGAGCGAAAGGTGATTCAGTACCAGAAATAGTGAAGCCACAGATGTATTTCTGGTACTGAATC ACCTTCTGCCTACTGCCTCGGA
LZTS1 sh-RNA2	TGCTGTTGACAGTGAGCGACGAAGACTTCTTCTACATCAATAGTGAAGCCACAGATGTATTGATGTAGAAGAAGT CTTCGCTGCCTACTGCCTCGGA
TXNIP sh-RNA1	TGCTGTTGACAGTGAGCGCAAGACTATATTTTGTACTTAATAGTGAAGCCACAGATGTATTAAGTACAAAATATA GTCTTTTGCCTACTGCCTCGGA
TXNIP sh-RNA2	TGCTGTTGACAGTGAGCGACCCAATGTACAGAATTATATATAGTGAAGCCACAGATGTATATATAATTCTGTACA TTGGGGTGCCTACTGCCTCGGA
CAMK2B sh-RNA1	TGCTGTTGACAGTGAGCGCACGGTGTTAGTTTGTAGGTAATAGTGAAGCCACAGATGTATTACCTACAAACTAAC ACCGTTTGCCTACTGCCTCGGA
CAMK2B sh-RNA2	TGCTGTTGACAGTGAGCGAAAGACCAGATGTGATTTGTTATAGTGAAGCCACAGATGTATAACAAATCACATCTG GTCTTGTGCCTACTGCCTCGGA
DAPK1 sh-RNA1	TGCTGTTGACAGTGAGCGAAAGCATGTAATGTTAATGTTATAGTGAAGCCACAGATGTATAACATTAACATTACA TGCTTCTGCCTACTGCCTCGGA
DAPK1 sh-RNA2	TGCTGTTGACAGTGAGCGCAAGAAGAGAATGACAATTCAATAGTGAAGCCACAGATGTATTGAATTGTCATTCTC TTCTTTTGCCTACTGCCTCGGA
UNC5B sh-RNA1	TGCTGTTGACAGTGAGCGCAAGGCTCAAAGAAGAAGAAAATAGTGAAGCCACAGATGTATTTTCTTCTTCTTTGA GCCTTATGCCTACTGCCTCGGA
UNC5B sh-RNA2	TGCTGTTGACAGTGAGCGCCGAGATGTATCTACTCATCAATAGTGAAGCCACAGATGTATTGATGAGTAGATACA TCTCGTTGCCTACTGCCTCGGA
SEMA6A sh-RNA1	TGCTGTTGACAGTGAGCGATAGGTGGTTGTTGTTGTTTTTAGTGAAGCCACAGATGTAAAAAACAACAACAACC ACCTACTGCCTACTGCCTCGGA
SEMA6A sh-RNA2	TGCTGTTGACAGTGAGCGCCACGACTTAATGTATGTTGTATAGTGAAGCCACAGATGTATACAACATACATTAAG TCGTGATGCCTACTGCCTCGGA
FOXO1 sh-RNA1	TGCTGTTGACAGTGAGCGACAGGAAAGTGATGTATAGTTATAGTGAAGCCACAGATGTATAACTATACATCACTT TCCTGCTGCCTACTGCCTCGGA
FOXO1 sh-RNA2	TGCTGTTGACAGTGAGCGACAGGTGGAGGTTGGTTTTGTATAGTGAAGCCACAGATGTATACAAAACCAACCTCC ACCTGGTGCCTACTGCCTCGGA

Table S2. Primer sequences used for qRT-PCR and sh-RNA amplify.

Gene symbol	Forward primer sequence（5'to3'）	Reverse primer sequence（5'to3'）
PTBP1	GTGTGCCATGGACGGCATT	TTTGCTGCAGAAGCCGAGT
ETV1	GTTTTTGCAGCCTTTCGCCT	ACTCTCGATGTTTCCCTGCG
ETV4	AAAAACAAGTCGGTGCGCTG	TTTCCGGGCGATTTCTGAGG
ETV5	CGACACTTGTGTTGTGCCTG	GGCTGGGTCATCAAGAAGGG
LZTS1	GGGAGGCCGAAAGTTTCTCT	AGTGTTTCCCGGTGTGTTCC
TXNIP	TATTGCAGGGCTTGGCAACT	TTGGGTGGCATGCAAGGTAT
CAMK2B	ACTTTGAGGCCTACGCGAAA	TGGATCGGCTTGCTGTTCTT
DAPK1	GGCGAGGGCTTCATTCTTCC	AAACTGTCACGCCTCACCAA
UNC5B	CTGTGCATGCAAATGCTGGAGG	TGTCTGTGTCGAAGTCACGG
SEMA6A	TCTTACAACACAGTGTATGGGCA	GCTGTCAGGTGAGTCAAGCA
FOXO1	GGCGTCCGTCCGTCCTTC	CTTAACTTCGCGGGGCCATC
sh-RNA	TGAACTCGAGAAGGTATATTGCTGTTGACAGTGAGCG	TCTCGAATTCTAGCCCCTTGAAGTCCGAGGCAGTAGGC
